# Deciphering the dose-dependent effects of thymoquinone on cellular proliferation and transcriptomic changes in A172 glioblastoma cells

**DOI:** 10.1371/journal.pone.0318185

**Published:** 2025-01-28

**Authors:** Rachana Pandey, Purushothaman Natarajan, Umesh K. Reddy, Wei Du, Cristian Sirbu, Moussa Sissoko, Gerald R. Hankins

**Affiliations:** 1 Department of Biology, West Virginia State University, Institute, WV, United States of America; 2 Department of Agriculture, Food, and Resource Sciences, University of Maryland Eastern Shore, Princess Anne, MD, United States of America; 3 Cancer Center, Charleston Area Medical Center, Charleston, WV, United States of America; 4 Institute for Academic Medicine, Charleston, WV, United States of America; 5 Katmai Oncology Group, Anchorage, Alaska, United States of America; Instituto do Cancer do Estado de Sao Paulo / University of Sao Paulo, BRAZIL

## Abstract

Glioblastoma multiforme (GBM), the most prevalent primary malignant brain tumor in adults, exhibits a dismal 6.9% five-year survival rate post-diagnosis. Thymoquinone (TQ), the most abundant bioactive compound in *Nigella sativa*, has been extensively researched for its anticancer properties across various human cancers. However, its specific anti-cancer mechanisms and pathways in glioblastoma remain to be completely elucidated. In this study, we assessed the impact of different TQ concentrations on the viability of A172 cells using WST-8 and Toluidine blue assays, followed by RNA sequencing (RNA-Seq) to identify differentially expressed genes (DEGs). We confirmed their expression levels through quantitative RT-PCR and performed Gene Ontology and Kyoto Encyclopedia of Genes and Genomes (KEGG) pathway enrichment analyses for these DEGs. RNA-seq revealed no significant gene expression changes at 2.5 μM and 5 μM TQ concentrations. However, at 25 μM and 50 μM, TQ significantly reduced cell viability dose-dependently. We identified 1548 DEGs at 25 μM TQ (684 up-regulated, 864 down-regulated) and 2797 DEGs at 50 μM TQ (1528 up-regulated, 1269 downregulated), with 1202 DEGs common to both concentrations. TQ inhibited key pathways such as PI3K-Akt signaling, calcium signaling, focal adhesion, and ECM-receptor interaction in A172 cells. It downregulated several potential oncogenes (e.g., *AEBP1*, *MIAT*) and genes linked to GBM proliferation and migration (e.g., *SOCS2*, *HCP5*) while modulating Wnt signaling and up-regulating tumor suppressor genes (e.g., *SPRY4*, *BEX2*). TQ also affected p53 downstream targets, maintaining p53 levels. This study elucidates the anti-cancer mechanisms of TQ in A172 GBM cells, underscoring its effects on multiple signaling pathways and positioning TQ as a promising candidate for innovative glioblastoma treatment strategies.

## 1. Introduction

In 2023, an estimated 1.9 million new cancer cases were projected in the United States, with over 600,000 fatalities anticipated, highlighting the ongoing challenge cancer poses to public health. Among these, brain and other nervous system tumors are particularly lethal, accounting for about 3% of all cancer-related deaths across genders [1]. Glioblastoma multiforme (GBM), the most prevalent primary malignant brain tumor in adults, with an incidence rate of 3.26 per 100,000 population, is especially notable for its aggressive nature [2–5]. Characterized by its rapid cell proliferation and poor differentiation, GBM represents a formidable challenge in oncology. The treatment protocol, which includes surgical resection followed by radiotherapy and adjuvant chemotherapy with temozolomide, remains the standard of care recommended by leading cancer networks and research [6, 7]. However, despite advancements in diagnostic and therapeutic strategies, the prognosis for GBM patients is grim, with a median overall survival of merely 13.5 months [5, 8]. This poor outcome is attributed to several factors, including high rates of resistance, the tumor’s invasive nature complicating surgical removal, adverse effects of treatments, an immunosuppressive tumor microenvironment, and the complexity of drug delivery across the blood-brain barrier [9, 10].

Recent interest in medicinal plants as potential anti-cancer agents has highlighted *Nigella sativa* Linn., known as Blackseed or Black cumin, as a significant subject of study [11]. Native to South and Southwest Asia, this annual flowering plant harbors a variety of bioactive compounds such as sterols, saponins, phenolic compounds, alkaloids, fatty acids, and volatile oils. Thymoquinone (TQ), a component of *Nigella sativa*’s volatile oil, is identified as the most pharmacologically active, exhibiting antimicrobial, antihistaminic, antidiabetic, anti-inflammatory, antioxidant, hypolipidemic, and anti-cancer properties [12]. Studies have demonstrated TQ’s cytotoxicity against diverse cancer cell lines, including breast adenocarcinoma, leukemia, lung cancer, colorectal carcinoma, pancreatic cancer, osteosarcoma, prostate cancer, and glioblastoma [13]. TQ’s anti-cancer actions involve multiple mechanisms, including modulating oxidative stress, interfering with DNA structure, and affecting cancer-signaling molecules and pathways [14]. While some mechanisms of TQ’s effects on glioblastoma cells are known, a comprehensive understanding of its molecular actions is still developing [15]. This study aims to analyze gene expression changes in A172 glioma cells treated with TQ using RNA-Seq to investigate the associated pathways of TQ’s differential gene expression effects.

## 2. Results

### 2.1. TQ treatment for 24 hours had no significant inhibitory effect on A172 cell viability

**Fig 1:**
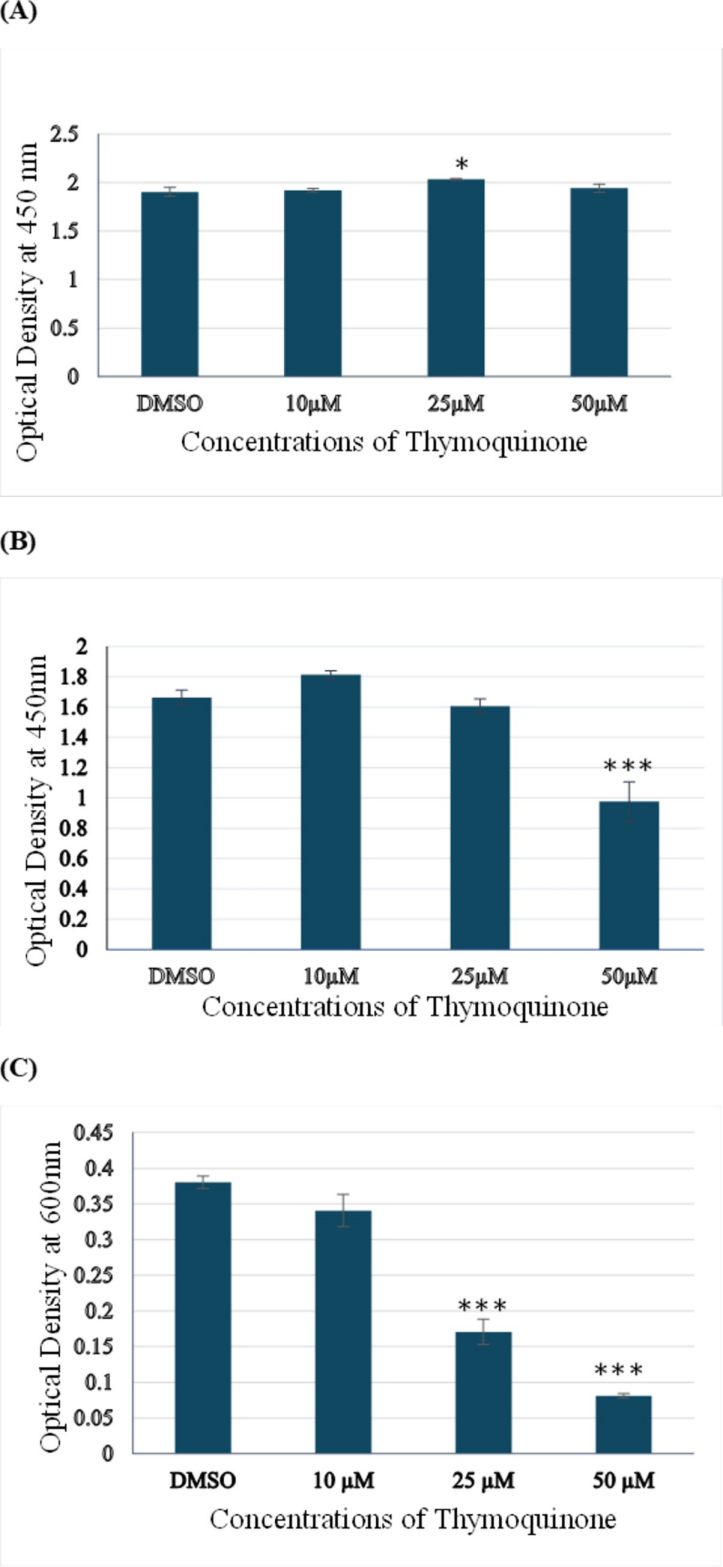
Effects of TQ on A172 cell viability. Viability of A172 cells after treatment with control, 10, 25, and 50 μM concentrations of TQ for 24 hours (A) and 48 hours (B and C). Cell viability was measured using WST-8 assay (A and B) and Toluidine Blue assay (C). The data are presented as the mean ±Standard Deviation. 0.1% DMSO was used for each treatment, including control. (n = 3 for each treatment, *p<0.05, **p<0.01, ***p<0.001).

We treated the A172 cells with increasing concentrations of thymoquinone (10, 25, and 50 μM) and treated for 24 hours as well as 48 hours. We then used WST-8 and Toluidine Blue assays to determine the effect of TQ on the numbers of viable A172 cells. As determined by the WST-8 assay in Fig 1, no significant growth inhibitory effect of TQ on A172 cells was observed across control, 10 μM, 25 μM, and 50 μM TQ treatment for 24 hours. If anything, TQ was observed to increase the numbers of viable cells in all the treatments, although any differences were small. Among the different concentrations, the proliferative response of A172 cells was higher at 25 μM. The 25 μM treatment significantly increased the number of viable cells when used for 24 hours. In contrast, the 10 and 50 μM treatments did not significantly increase the proliferation or number of A172 cells.

### 2.2. TQ treatment for 48 hours inhibited the viability of A172 cells in a dose-dependent manner

We further investigated the cytotoxic effects of 10, 25, and 50 μM TQ on A172 cells for 48 hours using WST-8 and Toluidine Blue assay. As shown in Fig 1, thymoquinone treatment for 48 hours displayed a dose-dependent reduction in viable cell numbers as analyzed by the WST-8 assay. The addition of 10 μM TQ did not significantly affect the viability of A172 cells. In contrast, treatment with 25 μM and 50 μM TQ for 48 hours resulted in a dose-dependent inhibition of cell viability, with statistically significant effects observed at 50 μM. WST-8 and WST-8 formazan have low toxicity, and the formazan is soluble, allowing further experiments to be conducted using the same cells (Dojindo Molecular Technologies, Inc., 2020). Following the WST-8 assay, the same cells were used directly for the toluidine blue cell viability assay. We simultaneously fixed and stained the cells with 100μl of toluidine blue staining solution and measured the absorbance at 600 nm. As shown in Fig 1, TQ decreased the number of viable cells dose-dependently. The growth inhibition at all concentrations (10 μM, 25 μM, and 50 μM) was statistically significant. The greatest decrease in the viability of A172 cells was observed at the TQ concentration of 50 μM (S1 Fig).

Overall, the results from both assays were consistent, except for the 10 μM treatment. The toluidine blue assay produced more dramatic results; however, cytotoxic assays like MTT and WST-8 are generally recognized for their higher sensitivity. WST-8, in particular, offers advantages such as low cytotoxicity and the production of a water-soluble formazan, which allows for subsequent experiments to be conducted on the same cells (Dojindo Molecular Technologies, Inc., 2020). For subsequent experiments, we used the results of the WST-8 assay as a reference. A limitation of the toluidine blue assay is the potential for experimenter error, such as rigorous washing of plates, which may inadvertently remove living cells adhered to the plate. This could impact readings, possibly due to altered cell adhesion. Our findings indicate that TQ inhibits the viability and/or proliferation of A172 cells at higher concentrations when exposed for an extended period (48 hours).

### 2.3. 2.5μM and 5 μM TQ for 24 hours did not cause differential gene expression in A172 cells

Initially, we treated A172 cells with control (the control group received 0.1% DMSO as a vehicle), 2.5 μM and 5 μM TQ for 24 hours, extracted and purified the RNA, and performed RNA-seq. In this case, we wanted to check for gene expression changes at concentrations of TQ that did not cause significant differences in the numbers of viable cells. Altogether, 352854966 raw reads were obtained, which were quality-filtered and mapped to the human reference genome (GRCh38.p13) https://www.gencodegenes.org/human [16]. Analysis using DESeq2 revealed no significant differential gene expression at these concentrations (2.5 μM, and 5 μM TQ). More details of this RNA-seq data are presented in S1 Table and the correlation heatmap of biological replicates are provided in S2 Fig.

### 2.4. TQ treatment for 48 hours resulted in differential gene expression in A172 cells

**Fig 2:**
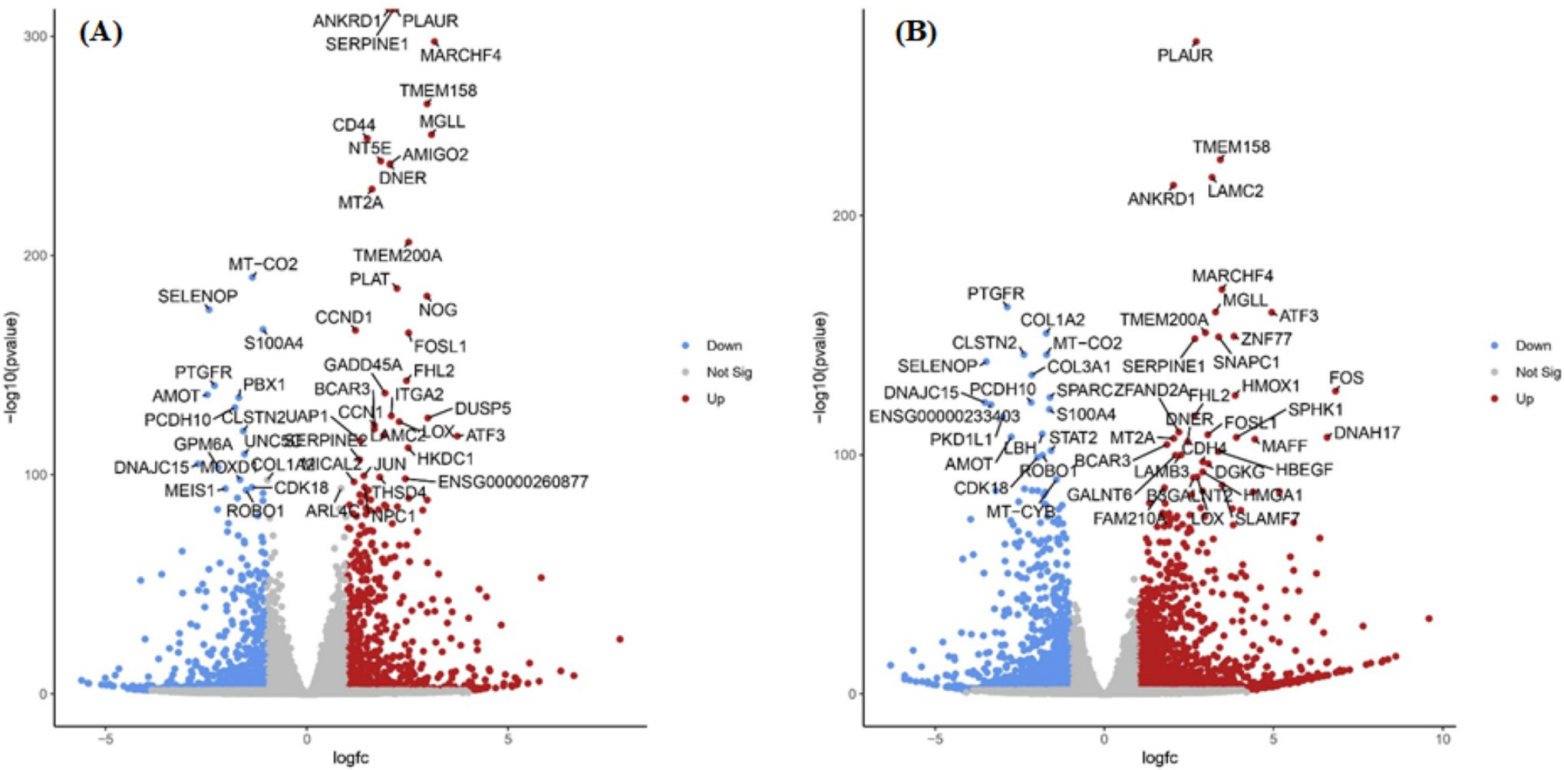
Volcano plots of DEGs in A172 cells after TQ treatment. Volcano Plots showing the distribution of all the DEGs (lfc > |1|, FDR < 0.05) identified in 25 μM TQ treatment for 48 hours in A172 cells (A) and 50 μM TQ treatment for 48 hours in A172 cells (B). Top 50 DEGs are labeled. Each dot represents a single gene.

**Fig 3:**
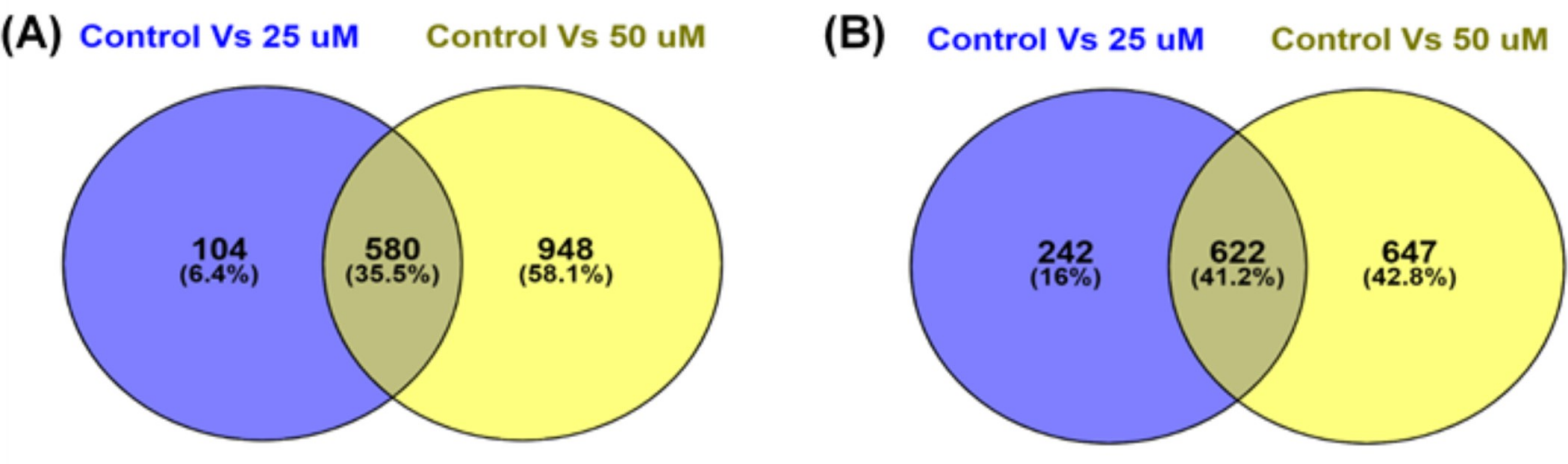
Venn diagrams of shared and unique up- and down-regulated genes. Venn-diagrams depicting the common and unique significantly up-regulated (A) and down-regulated (B) genes between Control vs 25 μM and Control vs 50 μM treatment.

**Fig 4:**
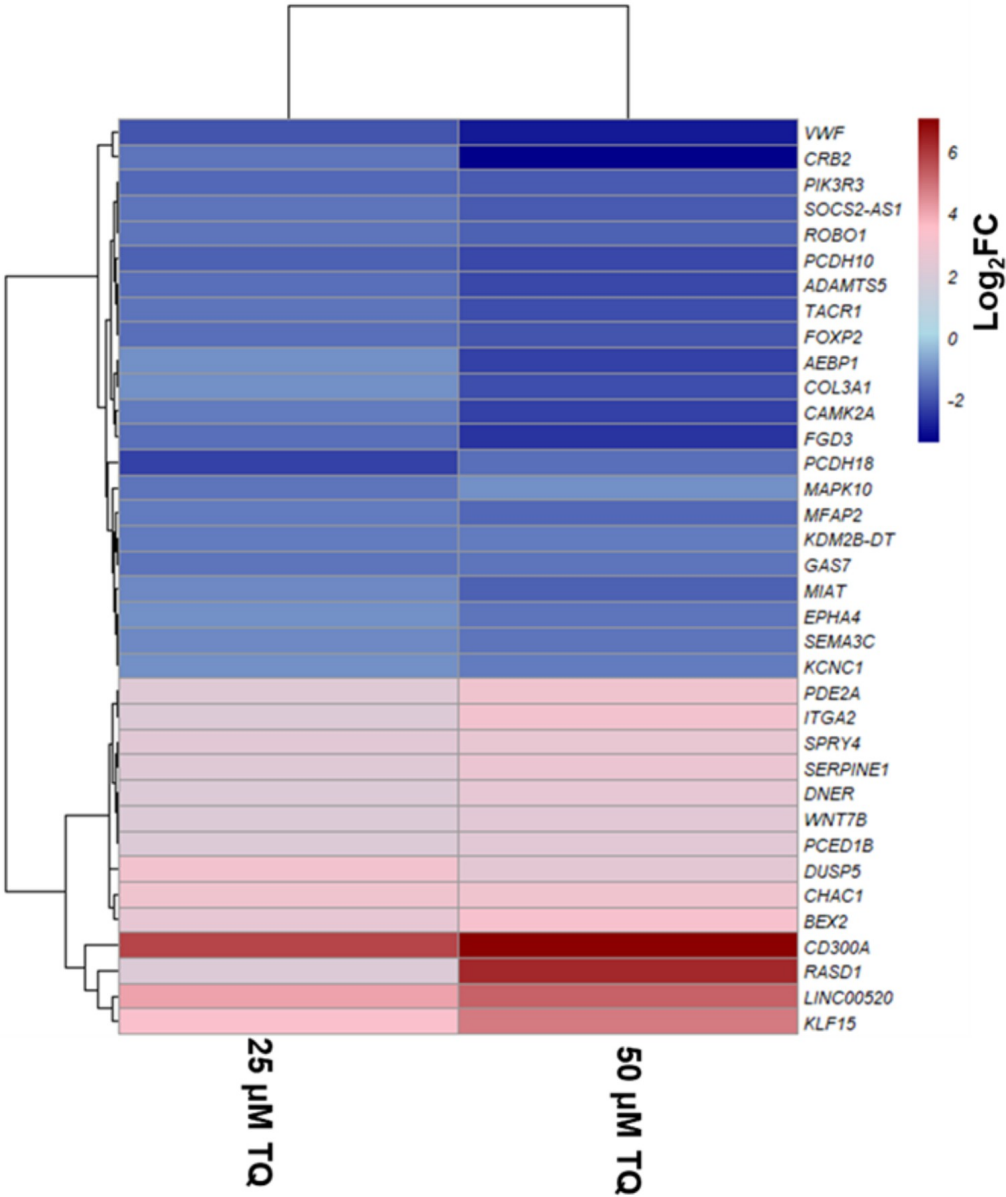
Heat map of significant DEGs. Heat map of significant DEGs in 25 μM and 50 μM TQ treatment showing the corresponding gene expressions. Blue color represents significantly downregulated genes, and red color represents significantly up-regulated genes.

A172 cells were treated with control (0.1% DMSO), 25 μM, and 50 μM TQ concentration for 48 hours, RNA was extracted, and RNA-sequencing was performed as described in the materials and method section. A total of 332,999,712 raw reads were primarily generated from RNA-seq. After removing the low-quality reads (Phred score QScore<30) and those containing sequencing adapters, 321,194,991 clean reads were retained. We then mapped the remaining clean reads to the reference human genome https://www.gencodegenes.org/human. The data quality, filtration, and mapping percentage details are presented in S2 Table. Using DESeq2 in RStudio version 3.0., we determined the genes with significant differential gene expression profiles between the control and different treatment conditions (Control vs. 25μM and control vs. 50 μM). Differential gene expressions were determined as the Log2 Fold-Change (Log2FC) greater than 1 or less than -1 with a false discovery rate (FDR)⩽0.05. Compared to the control, 1548 genes were differentially expressed in 25 μM TQ for 48 hours in which 684 genes were up-regulated and 864 were downregulated. These 1548 DEGs are visually represented in a volcano plot in Fig 2. In 50 μM TQ for 48 hours, 2797 genes were differentially expressed, of which 1528 were up-regulated, and 1269 were downregulated. Volcano plots for 2797 DEGs in 25 μM treatment are represented in Fig 2. We also determined the common significant DEGs in 25 and 50 μM treatment for 48 hours. In total, we found 1202 common differentially expressed genes. This includes 580 common significantly up-regulated genes and 622 common significantly downregulated genes. Similarly, 104 genes were found to be significantly up-regulated only on 25 μM for 48 hours. Also, 948 genes displayed significant upregulation solely on 50 μM treatment for 48 hours. Furthermore, 242 and 647 genes exhibited significant downregulation exclusively on 25 μM and 50 μM treatment for 48 hours, respectively. Venn diagrams representing common and unique significantly up-regulated genes and common and unique significantly downregulated genes on two treatment conditions are presented in Fig 3. A heat map of significant DEGs in 25 μM and 50 μM TQ treatment is presented in Fig 4.

### 2.5. KEGG pathway enrichment analysis

**Fig 5:**
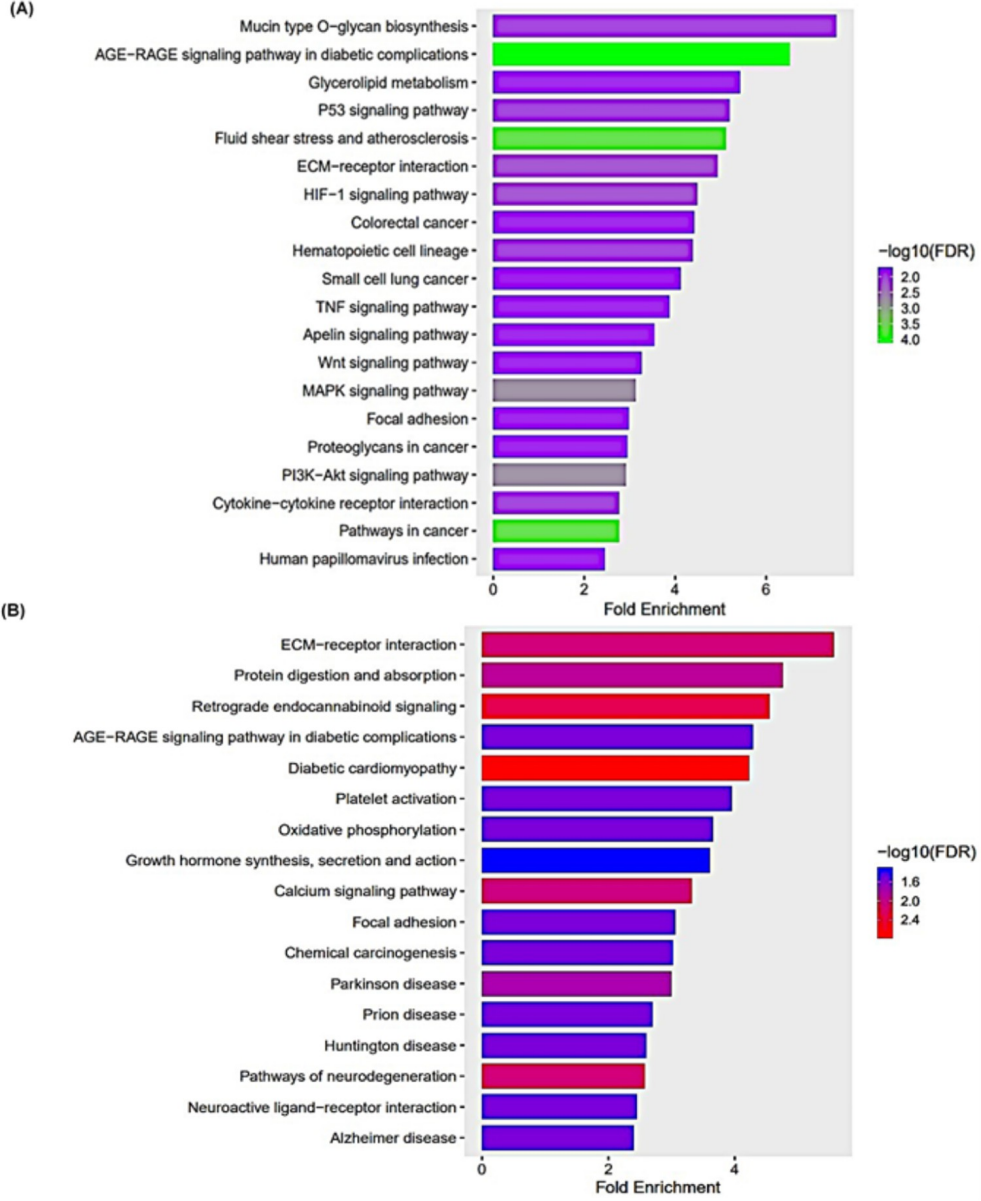
KEGG pathway enrichment of common DEGs. KEGG pathway enrichment bar plot for the common up-regulated (A) and down-regulated (B) genes in 25 and 50 μM TQ treatment for 48 hours on A172 glioblastoma cells. All common DEGs from the treatment were used for pathway analysis.

We performed KEGG pathway enrichment analysis for common significant DEGs in 25 μM and 50 μM TQ Treatment for 48 hours. We determined the important enriched biochemical, metabolic, and signal transduction pathways related to the activity of thymoquinone on human glioblastoma cells (A172). Using p<0.05 and a pathway size minimum of 15, we found that the common up-regulated genes were primarily enriched in 85 pathways. The top 20 highly enriched pathways are shown in Fig 5. In summary, those common up-regulated genes were enriched in several pathways known to be involved in tumorigenesis and the progression of glioblastoma. Some of these pathways are the P53 signaling pathway, ECM-receptor interactions, HIF-1 signaling pathway, TNF signaling pathway, WNT signaling pathway, MAPK signaling pathway, PI3-AKT signaling pathway, pathways in cancer, focal-adhesions, and cytokine-cytokine interactions.

Similarly, KEGG pathway analysis revealed that the common downregulated genes were enriched in the pathways related to ECM-receptor interaction, AGE-RAGE signaling pathway in diabetic complications, protein digestion and absorption, oxidative phosphorylation, calcium signaling pathway, focal adhesion, chemical carcinogenesis, and growth hormone synthesis, secretion, and action. The top 20 significantly enriched pathways are presented in Fig 5. The details of the enriched pathways, genes, and fold changes are presented in S3 and S4 Tables. The KEGG pathways with the identified genes from the current study are presented in S3–S7 Figs.

### 2.6. Gene ontology enrichment

Gene ontology enrichment analysis of common significant DEGs in 25 μM and 50 μM TQ treatment for 48 hours was conducted to explore those DEGs’ functions. The gene functions were categorized into three classes: biological process, cellular components, and molecular functions. The common up-regulated genes in the biological process category are the genes related to MAPK cascade, blood vessel development, cell migration, negative regulation of signal transduction, regulation of programmed cell death, and regulation of protein phosphorylation. Similarly, some cellular components highly enriched in those commonly downregulated genes include the intrinsic component of synaptic vesicle membrane, anchored component of membrane, extracellular matrix, and external encapsulating structure. Furthermore, the predominantly enriched molecular function terms were DNA binding, bending, insulin-like growth factor binding, extracellular matrix binding, growth factor activity, and cytokine activity.

**Fig 6:**
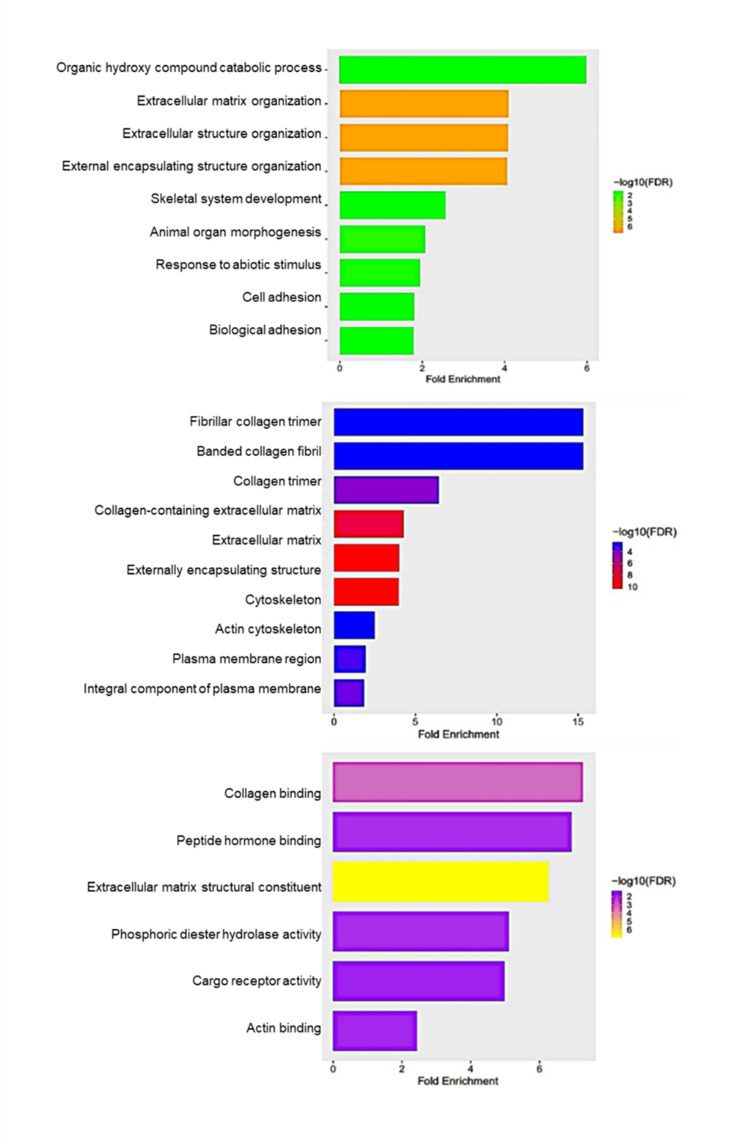
GO enrichment of common DEGs. GO enrichment for common DEGs in 25 and 50 μM TQ treatment for 48 hours on A172 glioblastoma cells. GO categories contain three domains: biological process (A), cellular Component (B), and molecular function (C). All common DEG from treatment were used for ontology enrichment.

Similarly, the common downregulated genes were significantly enriched in the biological process related to an extracellular matrix organization, extracellular structure organization, external encapsulating structure organization, cell adhesion, and focal adhesion. Similarly, the markedly enriched cellular components were fibrillar collagen trimer, collagen-containing extracellular matrix, actin cytoskeleton, extracellular matrix, external encapsulating structure, and integral component of postsynaptic density membrane. Similarly, the common downregulated genes are enriched in the molecular functions related to collagen binding, peptide hormone binding, and extracellular matrix structural constituents. The ten most significantly enriched biological processes, cellular components, and molecular functions are presented in Fig 6.

### 2.7. qRT-PCR verification

**Fig 7:**
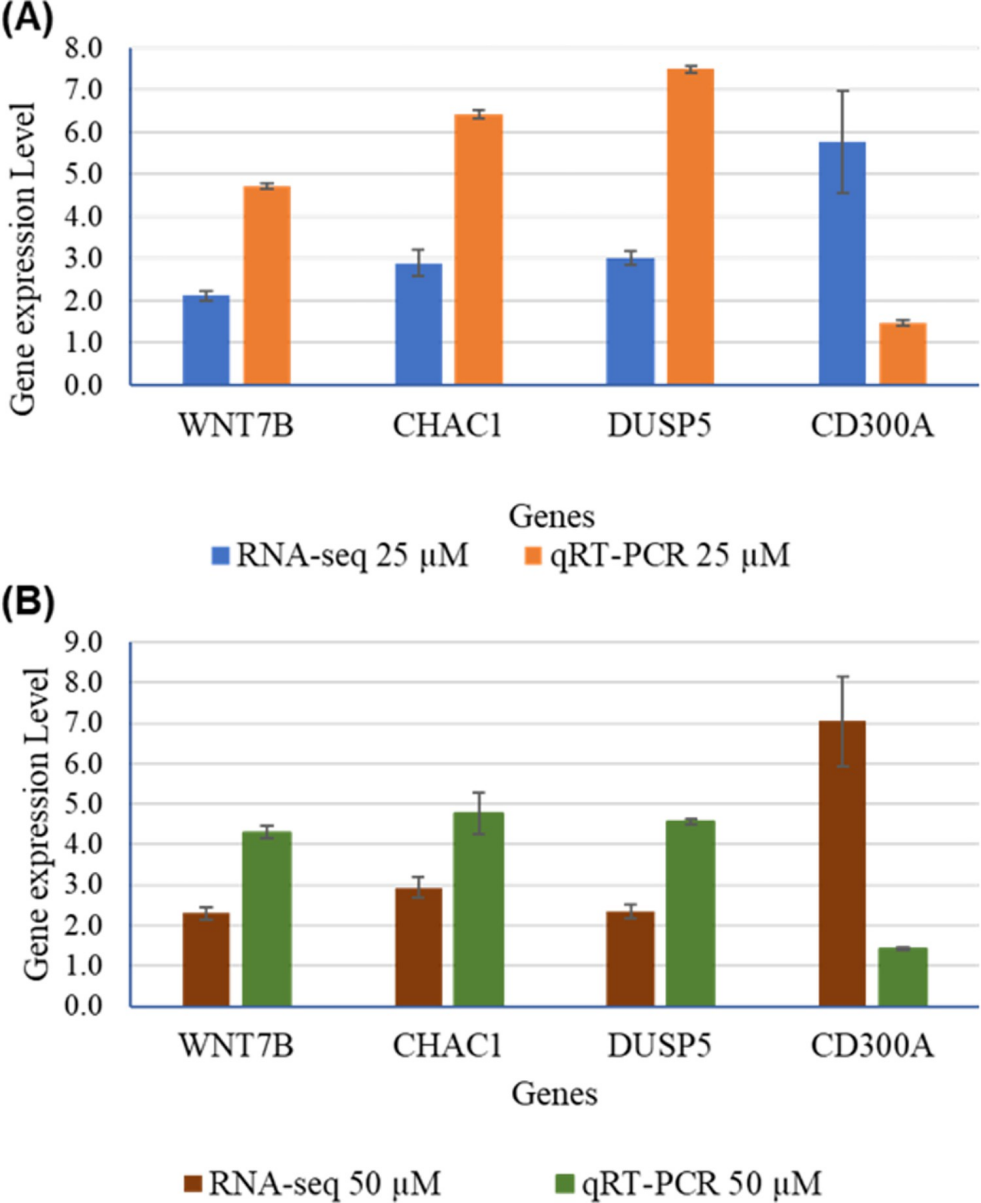
qRT-PCR validation of RNA-seq results. Validation of RNA-seq results by qRT-PCR in 25μM (A) and 50μM (B) TQ treatment condition. mRNA expressions of *WNT7B*, *CHAC1*, *DUSP5*, and *CD300A* were analyzed using qRT-PCR. The same RNA used for RNA-seq was used for qRT-PCR. RNA was extracted from A172 cells treated with control, 25 μM, and 50 μM TQ for 48 hours. The data are presented as the mean ±Standard Error of mean. (n = 3 for each treatment).

To further validate the gene expression results from Illumina RNA-Sequencing data, qRT-PCR was performed to investigate the mRNA expression of the genes *WNT7B*, *CHAC1*, *DUSP5*, and *CD300A* in TQ-treated A172 cells. Briefly, the same total RNA used for the RNA-seq was used for qRT-PCR. The details of the primers and sequences are presented in the S5 Table. All these genes are shown to be significantly up-regulated by qRT-PCR result, consistent with the RNA-seq findings. Gene expression folds change in NGS and qRT-PCR is presented in Table 1. qRT-PCR results are shown graphically in Fig 7. Additionally, melt curve analyses, demonstrating assay specificity are presented in S8 Fig. These findings demonstrate that the expression pattern of all genes is consistent with Illumina RNA-Sequencing data, confirming the validity of our experimental results.

**Table 1:** The fold change of some important genes over different treatment conditions in RNA-seq and qRT-PCR.

Genes	RNA-Seq				qRT-PCR			
	25 μM		50 μM		25 μM		50 μM	
	Fold Change	P-adj	Fold Change	P-adj	Fold Change	P-adj	Fold Change	P-adj
*WNT7B*	2.123	4.72E-76	2.2909	5.01E-48	4.7148	0.0003	4.2974	0.0005
*CHAC1*	2.897	9.1E-19	2.9376	9.89E-31	6.4202	0.0264	4.7567	0.0637
*DUSP5*	3.006	9.31E-124	2.3411	1.55E-47	7.4982	0.000006	4.5518	0.00022
*CD300A*	5.776	1,11E-05	7.0351	4.28E-09	1.4704	0.000281	1.4261	0.00049

## 3. Discussion

In our study investigating the impact of thymoquinone (TQ) on A172 glioma cell viability, we observed a dose-dependent inhibition of cell proliferation at higher concentrations, particularly after 48 hours of treatment. These results are consistent with existing literature, such as Ballout and Gali-Muhtasib, 2020 [17], who reported similar time and concentration-dependent effects of TQ in neuroblastoma cells. Similarly, Kolli-Bouhafs [18] and his colleagues reported 50μM TQ treatment to reduce migration, adhesion, and invasion of both human glioblastoma cell lines, U-87 MG and CCF-STTG1 cells, in vitro. Also, Guler et al. 2021 [19] noted the cytotoxicity of TQ in rat C6 glioma cells in a concentration-dependent manner. Decreased numbers of viable U-251MG after TQ treatment have also been reported by Altundağ et al., 2023 [20] and Mai et al., 2021 [21]. The dual role of TQ in modulating cell proliferation, as seen in our findings and echoed by Fatfat et al. (2021) [22], highlights its complex biological activity. Our reliance on the WST-8 assay for its reliability, as also suggested by Eid et al. (2023) [23], helped mitigate potential experimental errors common in assays like Toluidine Blue, as discussed by Adilovic et al. (2020) [24].

Cell viability remained unaffected at 24 hours of TQ treatment and showed no significant changes at concentrations up to 25 μM after 48 hours. However, cell adhesion was notably reduced at 25 μM, consistent with the observed downregulation of extracellular matrix (ECM)-related genes. Furthermore, TQ significantly disrupted the ECM-receptor interaction pathway in A172 glioblastoma cells, highlighting its potential impact on adhesion and migration. The ECM components form the cellular microenvironment and is often overexpressed in glioma cells [25]; components such as hyaluronic acid, brevican, tenascin-C, fibronectin, thrombospondin, and specific integrins and receptors are known to promote cell adhesion and migration [26]. These components, particularly HA and collagens, also act as barriers that limit drug diffusion and penetration into tumors [27]. TQ treatment’s downregulation of several ECM components suggests potential therapeutic effects, such as decreased cell adhesion, impaired cancer cell migration, and hindered invasion through the basement membrane.

Further, we noted that TQ inhibited the calcium signaling pathway in A172 glioblastoma cells, as evidenced by the downregulation of genes associated with this pathway following treatment with 25 and 50 μM TQ for 48 hours. Calcium signaling, crucial in controlling diverse cellular functions and linked with the progression of GBMs and other cancers [28, 29], saw notable gene expression changes. *CAMK2A*, a key kinase in Ca2+-induced signaling and cell cycle progression, was significantly downregulated, aligning with findings by Takemoto‐Kimura et al. (2017) [30] on its role in proliferation. Studies by Wang et al. (2022) [31] and Yu et al. (2021) [32] further emphasize its impact on glioma cell functions. The *TACR1* gene, encoding a receptor linked to various cancers [33], also showed decreased expression, highlighting TQ’s potential in cancer cell apoptosis. Moreover, the reduced expression of *GRM1*, involved in cell cycle arrest and apoptosis in glioma [34, 35], and the downregulation of *PLCG2*, known for its overexpression in glioma and role in intracellular Ca2+ signaling [36, 37], further underscore TQ’s multifaceted impact on glioblastoma cell signaling and tumor progression.

In our study, KEGG pathway analysis indicated that higher concentrations of Thymoquinone (TQ) (50 μM) significantly downregulated genes in the PI3K-Akt signaling pathway in A172 glioblastoma cells, involving 18 differentially expressed genes like *LPAR2*, *FGF10*, *COL9A3*, *PDGFB*, *IL2RB*, *IL7*, *PDGFRB*, *ITGB6*, *PPP2R2B*, *COL1A2*, *THBS3*, *PDGFD*, *GNG7*, *ERBB4*, *COL4A5*, *LAMA2*, *COL6A6*, *ITGA1*. This pathway, crucial in processes like proliferation, differentiation, migration, metabolism, and survival, is often dysregulated in GBM due to *RTK* gene mutations and PI3K pathway activation. Interestingly, we observed the upregulation of *RASD1*, a gene that inhibits glioma cell migration and invasion by deactivating the AKT/mTOR signaling pathway [38]. This might contribute to the observed inhibition of the AKT pathway. Additionally, *PPP2R2B*, often downregulated and methylated in GBM, was also downregulated in TQ-treated cells, correlating with shorter survival in GBM patients [39].

Treatment with 25 and 50 μM thymoquinone (TQ) for 48 hours up-regulated the p53 signaling pathway in A172 glioblastoma cells based on the RNA-Seq data without directly altering p53 levels. This suggests TQ modulates the p53 pathway by influencing the expression of its downstream target genes. Specifically, TQ up-regulated genes like *PMAIP1 (NOXA)*, *GADD45A*, *BBC3 (PUMA)*, and *DUSP5*. *PMAIP1* and *BBC3*, transcription targets of p53 involved in DNA damage-induced apoptosis [40–43], are pro-apoptotic proteins of the Bcl-2 family, which, when activated, bind and inhibit anti-apoptotic proteins. *GADD45A* is rapidly induced in response to DNA damage [44, 45], and studies have shown its importance in apoptosis induction by anti-cancer agents [46–48]. *DUSP5*, a dual-specificity phosphatase, deactivates protein kinases and its overexpression has been linked to suppressed growth in various cancer cells [49]. The increase in proapoptotic proteins and decrease in anti-apoptotic proteins like Bcl-XL in TQ-treated cells suggest TQ may enhance mitochondrial membrane permeability, potentially activating the intrinsic pathway of apoptosis in glioblastoma cells.

In our study, TQ treatment of A172 glioblastoma cells with 25 and 50 μM concentrations for 48 hours significantly up-regulated two candidate tumor suppressor genes: Sprouty RTJ Signaling Antagonist 4 (SPRY4) and Brain Expressed X-Linked 2 (BEX2). SPRY4, as a negative regulator of MAPK activation, its ectopic expression has been shown to inhibit proliferation and migration in GBM cells, suggesting its role as a tumor suppressor [50]. *BEX2*, part of the BEX gene family, was found to be silenced in U-87 and primary glioma cell lines, with its re-expression increasing sensitivity to chemotherapy-induced apoptosis and demonstrating tumor suppressor effects [51]. However, some studies show contrasting results about *BEX2*, such as its high expression in glioma tissue and its role in inhibiting glioma cell migration and invasion by affecting β-catenin levels [52]. These findings suggest that TQ may play a role in modulating tumor suppressor gene expression, contributing to its potential therapeutic effects in glioblastoma treatment.

In our study on A172 glioblastoma cells, TQ regulated the expression of key components and regulators of the Wnt signaling pathway by increasing *Wnt7B* expression(2.2-fold) and decreasing *WNT6* expression (1.6-fold). The Wnt pathway plays a crucial role in processes like cell proliferation, apoptosis, migration, and invasion. Varied expression levels of Wnt pathway components in gliomas have been observed, such as elevated *WNT3A* and 5A and decreased *WNT7B* [53], and the oncogenic role of overexpressed WNT6 [54]. In our study, Phosphodiesterase 2A (*PDE2A*) was up-regulated, a gene known to suppress Wnt/β-catenin signaling in glioma stem-like cells by modulating cAMP accumulation and GSK-3β phosphorylation [55]. Additionally, TQ downregulated Sema3C, an overexpressed gene in most GBMs that activates the canonical Wnt pathway [56]. Our findings suggest that TQ modulates Wnt signaling by altering the expression of its core components (*WNT7B* and *WNT6*) and regulators (*PDE2A* and *Sema3C*), thereby potentially downregulating this oncogenic pathway and contributing to its anti-cancer mechanism in vitro.

Furthermore, TQ treatment led to the upregulation of genes like *CHAC1* and *DNER*, which are known for their roles in GBM development. *CHAC*, typically downregulated in GBM cell lines, was identified as a key gene up-regulated by TMZ treatment, enhancing glioma apoptotic death and inhibiting Notch3-mediated pathways [57]. *DNER*, a noncanonical Notch ligand, was found to suppress glioma growth by inhibiting the oncogene *TOR4A* [58] and hindered the growth and induced differentiation of GBM-derived neurospheres [59]. Conversely, TQ treatment resulted in the downregulation of several genes overexpressed in GBM cells, including potential oncogenes like *AEBP1*, *MIAT*, *GHR*, *LMO1*, *ELF3* [60–64], and genes involved in tumor proliferation and migration such as *EPHA4*, *COL3A1*, *PCDH10*, *ROBO1*, *ADAMTS5*, *PCDH18*, *ST8SIA1* [65–70]. TQ downregulated the expression of Von Willebrand Factor (*VWF*), a highly adhesive procoagulant molecule, by more than 2-fold. It is a prognostic factor in GBM [71]. We also observed the significant downregulation of crumbs homolog 2 (*CRB2*) in both 25 μM (1.4 fold) and 50 μM (3.4 fold). The lentivirus knockdown of CRB2 suppressed the GBM proliferation, migration, and invasion. Increased level of *CRB2* increased the phosphorylation of IκB-kinase α (*IKKα*) consequently activating NF-κB via reduction of Ikβ protein [72]. Additionally, TQ significantly downregulated genes associated with poor GBM prognosis, including *PCSK5*, *KCNC1*, *MXRA5*, *SEMA3C*, *MFAP2*, *MTERF2*, *KDM2B*, *FOXP2* [73–79], indicating TQ’s potential in targeting key molecular pathways in GBM.

## 4. Materials and methods

### 4.1. Cell culture and treatment

The glioblastoma cell line, A172 (ATCC #CRL-1620, Manassas, VA, USA) were cultured in Dulbecco’s modified Eagle medium (DMEM, ATCC, Cat #30–2002) supplemented with 10% (v/v) Fetal bovine serum (FBS, Atlas Biologicals, Fort Collins, CO, Cat #F-0500-A) and an antibiotic-antimycotic mixture: of penicillin (10 000 U/mL), streptomycin (10mg/ mL), and amphotericin (25μg/mL) (Millipore Sigma Life Sciences, Burlington, MA, Cat #A5955). Cells were maintained at 37°C with 5% CO2 and passaged using trypsin-EDTA (ATCC, Manassas, VA). Thymoquinone (TQ, Millipore Sigma, Cat #274666) was dissolved in DMSO and diluted in DMEM to achieve the final working concentrations (0–50 μM) with 0.1% DMSO for all treatments. A final concentration of 0.1% DMSO was used to ensure solubility of TQ without affecting cell viability, as higher concentrations of DMSO (e.g., 1–5%) are known to be cytotoxic. After overnight attachment, cells were treated with different concentration of thymoquinone for 48 hours.

### 4.2. Toluidine blue staining

A172 cells were seeded into 96 well plates, incubated overnight, and treated with 0, 10, 25, and 50μM concentrations of TQ for 48 hours. After treatments, cells were washed once with phosphate-buffered saline (PBS) (ATCC) and stained with 100μl of toluidine blue solution (1% toluidine blue (LabChem Inc, Zelienople, PA) and 1% borax (LabChem)) for 20 minutes at room temperature. The staining solution was discarded and the plate was washed and dried. The next day, absorbance at 600nm was measured using SpectraMax iD3 Multi-Mode Microplate Reader (Molecular Devices, San Jose, CA).

### 4.3. WST-8 assay

The WST-8 assay was performed using the Cell Counting Kit-8 (CCK-8) following the manufacturer’s protocol (Dojindo Laboratories, Gaithersburg, MD). A172 cells were seeded at a density of 5,000 cells per well in 100 μL of DMEM in 96-well plates and incubated overnight at 37°C in a humidified 5% CO₂ atmosphere. The next day, the cells were treated with control (0.1% DMSO), 10 μM, 25 μM, and 50 μM thymoquinone (TQ). After 48 hours of treatment, 10 μL of CCK-8 solution was added to each well and incubated for 3 hours. Following incubation, absorbance was measured at 450 nm using a SpectraMax iD3 Multi-Mode Microplate Reader (Molecular Devices, San Jose, CA).

### 4.4. RNA extraction, library preparation, and RNA-sequencing

A172 cells were seeded in 60mm culture dishes and incubated overnight. After 24 hours of incubation, the cells were treated with 25 and 50 μM concentrations of thymoquinone for 48 hours. Total RNA was extracted using Qiagen RNeasy Total RNA Isolation Kit (QIAGEN, Valencia, CA). The concentration and integrity of RNA samples were evaluated using a Nanodrop spectrophotometer (Thermo Fisher Scientific) and 0.7% % agarose gel electrophoresis and quantified using Qubit 3.0 (Thermo Fisher Scientific, USA). RNA Seq-Libraries were prepared using 1 μg of total RNA from each of the three biological replicates following the NEBNext®Ultra™ RNA Library Prep Kit for Illumina® (New England Biolabs Inc., Ipswich, MA, USA, Cat #E7770S/L, #E7775S/L). The final library quality and insert size were determined using a bioanalyzer (Invitrogen, USA), and the library was quantified using a Qubit fluorometer (Invitrogen, USA). The library was diluted to 4nM concentration and sequenced using Illumina’s NextSeq 500 platform with 150bp paired-end sequencing chemistry.

### 4.5. Differential gene expression and pathway analysis

The raw data was obtained in FASTQ format. The quality of the raw data was assessed using the tool FastQC. For quality control, Trimmomatic (v0.30) was used to remove low-quality bases (Quality Score <30) and those containing the adapter sequences (Bolger et al., 2014). The reference human genome sequences (GRCh38.p13) and the gene annotation files were then downloaded from gencode website (https://www.gencodegenes.org/human/). STAR (version 2.7.3a) [80] was used to index the reference human genome sequences in order to create the necessary data structures for efficient alignment. After creating the reference genome indexed file, the clean data were aligned to them via STAR aligner [80]. Using the SAMTools, the aligned transcriptomic data in SAM format was converted into BAM format. The aligned BAM file was indexed using the SAMtools. The read count table was generated from the BAM alignment file and general feature format (GFF) of genome annotation using the HTSeq R package [81]. The Differentially expressed genes (DEGs) among different experimental pair-wise combinations were identified using the DESeq2 [82]. The DEGs were filtered based on the minimum Log2 Fold-Change (Log2FC) greater than 1 or less than -1 with a false discovery rate (FDR)⩽0.05. GO and Pathway Enrichment analyses were performed using ShinyGO [83]. Similarly, heatmap was generated using statistical package pheatmap in RStudio, venn-diagram using Venny 2.1, and volcano plot was generated using online tool Galaxy Bioinformatics.

### 4.6. Real-time reverse transcription polymerase chain reaction (qRT-PCR)

Total RNA was isolated and purified from cells treated with control, 10, 25, and 50μM concentrations of TQ for 48 hours. Then, 500ng of total RNA was reverse transcribed into cDNA using random primers in a ImProm-II Reverse Transcription System (Promega, Madison, WI) following the manufacturer protocol. The RT reaction was carried out in a total volume of 20 μl. The quality and quantity of cDNA were assessed using a Nanodrop spectrophotometer (Thermo Fisher Scientific). cDNA from 27ng of RNA template and 100μM of each target genes primers was used. The mRNA expression level of the target gene was normalized to 18S rRNA mRNA expression. Fold change in the mRNA expression relative to the control was calculated using the comparative method based on 2-ΔΔCt values with ABI SDS v1.2.3 software [84].

### 4.7. Statistical analysis

To test for significance, a one-way analysis of variance (ANOVA) was used. This test was used to determine whether there were statistically significant differences among the means of control and treatment groups. Data were presented as means + SEM. The confidence interval was 95%, and the P<0.05 was considered significant. For the multiple comparisons of the means, Tukey HSD post hoc test was conducted on R Studio.

## 5. Conclusions

The findings of this study highlight the dose-dependent effects of Thymoquinone (TQ) on glioblastoma multiforme (GBM) cells. At higher concentrations (25 μM and 50 μM), TQ significantly inhibited cell viability and modulated the expression of key genes, as evidenced by RNA sequencing. Pathway enrichment analysis revealed that TQ affects critical cancer-related pathways, including PI3K-AKT signaling, calcium signaling, focal adhesion, ECM-receptor interaction, and the p53 signaling pathway. TQ also influenced Wnt signaling components, leading to potential anti-cancer effects such as impaired migration, adhesion, and proliferation.

Moreover, TQ’s effects extended to the modulation of oncogenes and tumor suppressor genes, suggesting its therapeutic potential in targeting GBM. While this study provides valuable insights into TQ’s mechanisms of action, future research should validate these findings through functional studies and expand investigations to additional glioblastoma cell lines and in vivo models. The ability of TQ to penetrate the blood-brain barrier, coupled with its demonstrated molecular effects, underscores its promise as a therapeutic agent for GBM.

## Supporting information

S1 Fig GBM cells treated with varying thymoquinone concentrations. GBM cells with various concentrations of Thymoquinone (A) Control- 0 μM (without TQ) (B) 10 μM (C) 25 μM (D) 50 μM.
(TIF)

S2 Fig Pearson correlation heatmap of RNA-seq samples. Pearson correlation heatmap displaying the correlation coefficients between RNA-seq samples treated with varying concentrations of thymoquinone (TQ) and untreated controls. Samples are labeled according to their treatment groups and biological replicates. The color gradient ranges from white (low correlation) to blue (high correlation), with light grid lines for clarity. High intra-group correlation highlights the treatment-induced gene expression patterns across replicates.
(TIF)

S3 Fig ECM-receptor interaction pathway in TQ-treated A172 cells. ECM-receptor interaction pathway in response to 25 and 50 μM TQ treatment for 48 hours in A172 glioblastoma cells. Red boxes represent upregulated genes, while green boxes show downregulated genes. The intensity of the color reflects the level of change. Gray boxes indicate genes present in the pathway but not detected in the data. Arrows show the connections between the genes and their interactions in the pathway.
(TIF)

S4 Fig Focal adhesion pathway in TQ-treated A172 cells. Focal adhesion pathway in response to 25 and 50 μM TQ treatment for 48 hours in A172 glioblastoma cells. Red boxes represent upregulated genes, while green boxes show downregulated genes. The intensity of the color reflects the level of change. Gray boxes indicate genes present in the pathway but not detected in the data. Arrows show the connections between the genes and their interactions in the pathway.
(TIF)

S5 Fig Calcium signaling pathway in TQ-treated A172 cells. Calcium signaling pathway in response to 25 and 50 μM TQ treatment for 48 hours in A172 glioblastoma cells. Red boxes represent upregulated genes, while green boxes show downregulated genes. The intensity of the color reflects the level of change. Gray boxes indicate genes present in the pathway but not detected in the data. Arrows show the connections between the genes and their interactions in the pathway.
(TIF)

S6 Fig PI3K-AKT signaling pathway in 50 μM TQ-treated A172 cells. PI3K-AKT signaling pathway in response to 50 μM TQ treatment for 48 hours in A172 glioblastoma cells. Red boxes represent upregulated genes, while green boxes show downregulated genes. The intensity of the color reflects the level of change. Gray boxes indicate genes present in the pathway but not detected in the data. Arrows show the connections between the genes and their interactions in the pathway.
(TIF)

S7 Fig P53 signaling pathway in TQ-treated A172 cells. P53 signaling pathway in response to 25 and 50 μM TQ treatment for 48 hours in A172 glioblastoma cells. Red boxes represent upregulated genes, while green boxes show downregulated genes. The intensity of the color reflects the level of change. Gray boxes indicate genes present in the pathway but not detected in the data. Arrows show the connections between the genes and their interactions in the pathway.
(TIF)

S8 Fig Melt curve analysis and results table for qRT-PCR validation. **(A)** Melt curve (derivative reporter) displaying amplification specificity for the housekeeping gene 18s rRNA (red) and the genes of interest: *DUSP5* (green), *CD300A* (cyan), *CHAC1* (light green), and *WNT7B* (yellow). The sharp peaks indicate single, specific amplicons for each gene, confirming specificity. **(B)** Melt curve (normalized reporter) showing fluorescence intensities normalized across the samples for consistent data interpretation. The normalized curves reaffirm the specificity of the amplified products. **(C)** Results table summarizing the qRT-PCR data, including Ct values, fold changes, and melting points (Tm) for each gene under different conditions (control, 25 μM, and 50 μM TQ treatments). Housekeeping gene 18s rRNA served as internal control. The melting temperatures for each gene align with their respective peaks in the melt curve analyses, confirming assay efficiency and specificity.
(TIF)

S1 Table Data quality and mapping summary. Summary of data quality, filtration, and mapping of A172 glioblastoma cells treated with control, 2.5 μM, and 5 μM TQ for 24 hours.
(XLSX)

S2 Table Data quality and mapping summary. Summary of Data Quality, filtration, and mapping of A172 glioblastoma cells under 25 μM and 50 μM treatment for 48 hours.
(XLSX)

S3 Table KEGG pathway enrichment for common downregulated genes. The KEGG pathway enrichment table for the common downregulated genes in 25 and 50 μM TQ treatment for 48 hours on A172 cells.
(XLSX)

S4 Table KEGG pathway enrichment for common upregulated genes. The KEGG pathway enrichment table for the common upregulated genes in 25 and 50 μM TQ treatment for 48 hours on A172 cells.
(XLSX)

S5 Table qRT-PCR primer sequences. Target genes and their respective primer sequences for qRT-PCR.
(XLSX)
