# Supplementary figures and images for: Deciphering the dose-dependent effects of thymoquinone on cellular proliferation and transcriptomic changes in A172 glioblastoma cells
Source: PLoS One. 2025 Jan 28;20(1):e0318185. doi: 10.1371/journal.pone.0318185 (PMC11774404; doi:10.1371/journal.pone.0318185)

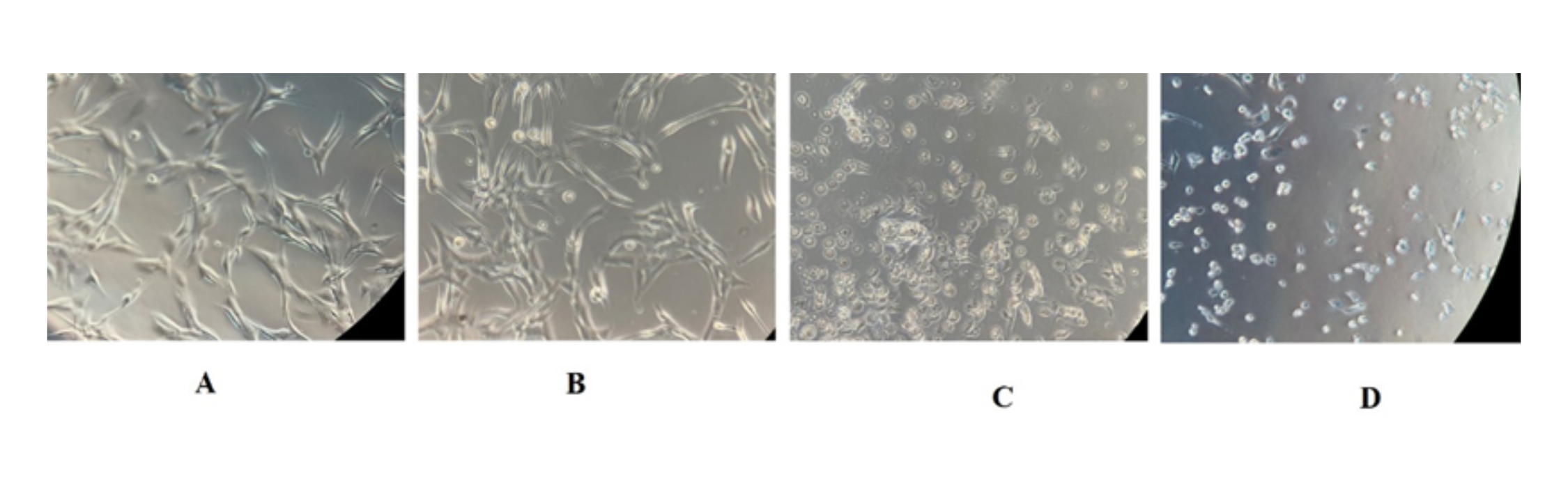

Supplement: S1 Fig — GBM cells with various concentrations of Thymoquinone (A) Control- 0 μM (without TQ) (B) 10 μM (C) 25 μM (D) 50 μM. (TIF) [file pone.0318185.s001.tif]

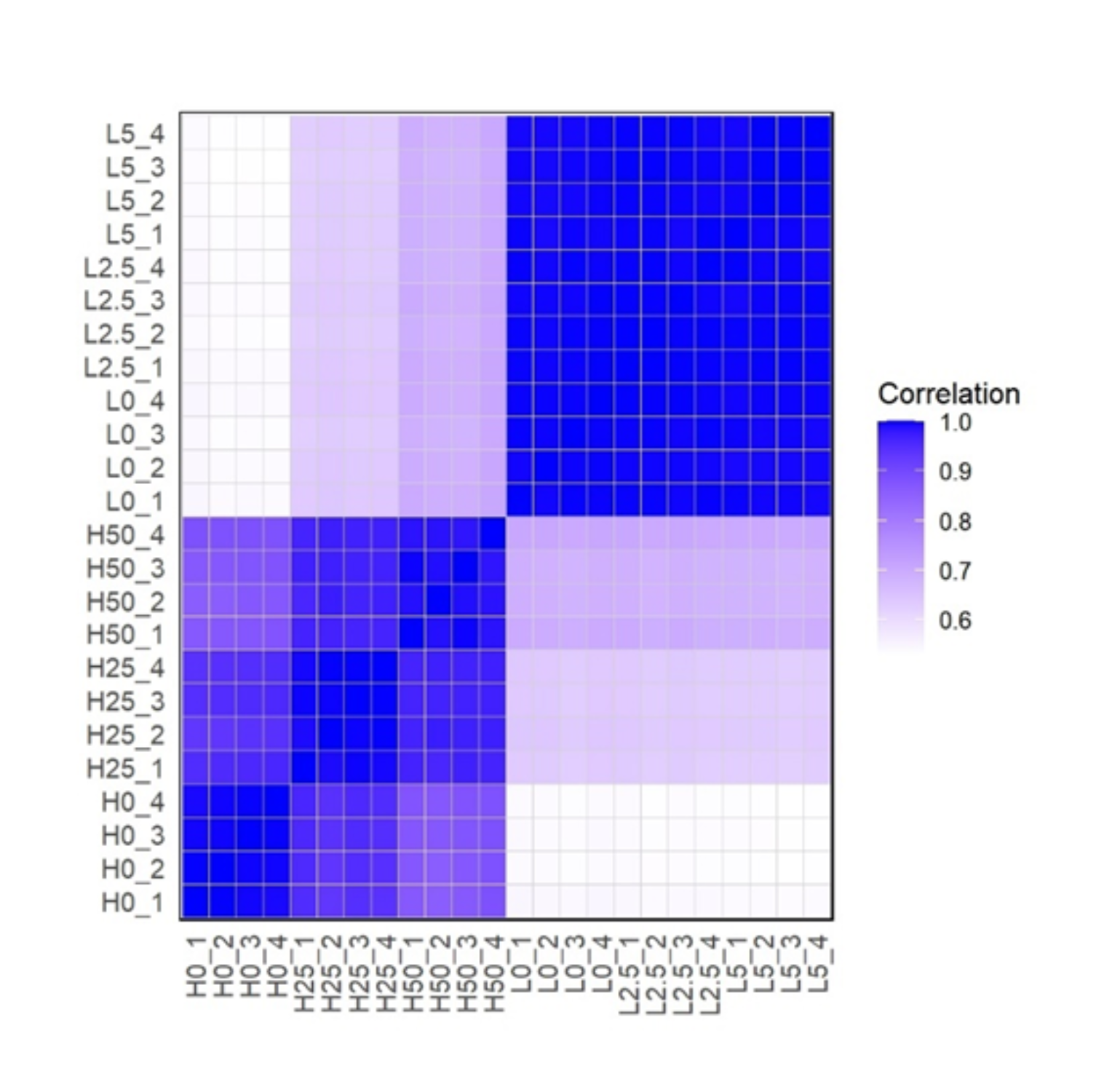

Supplement: S2 Fig — Pearson correlation heatmap displaying the correlation coefficients between RNA-seq samples treated with varying concentrations of thymoquinone (TQ) and untreated controls. Samples are labeled according to their treatment groups and biological replicates. The color gradient ranges from white (low correlation) to blue (high correlation), with light grid lines for clarity. High intra-group correlation highlights the treatment-induced gene expression patterns across replicates. (TIF) [file pone.0318185.s002.tif]

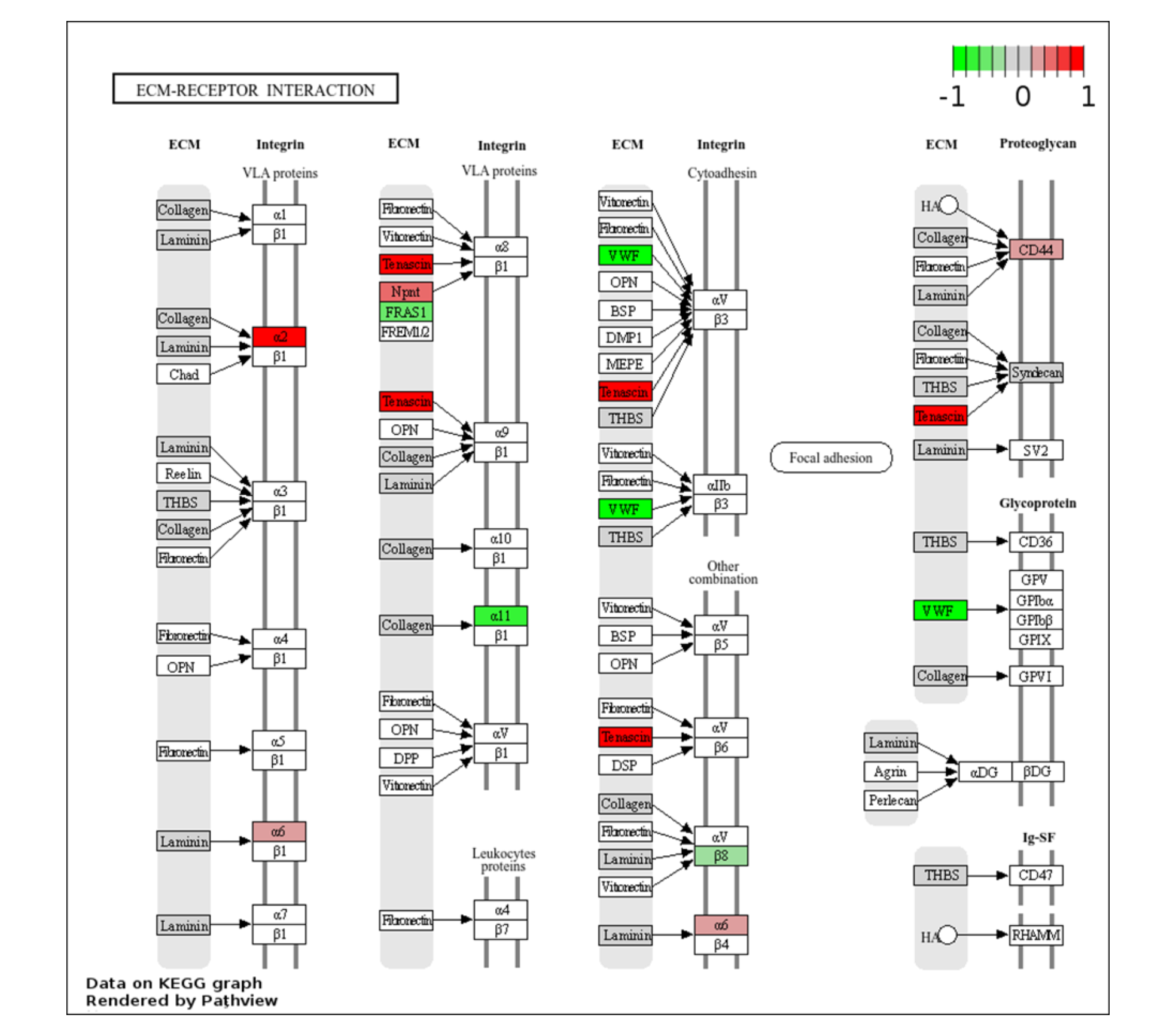

Supplement: S3 Fig — ECM-receptor interaction pathway in response to 25 and 50 μM TQ treatment for 48 hours in A172 glioblastoma cells. Red boxes represent upregulated genes, while green boxes show downregulated genes. The intensity of the color reflects the level of change. Gray boxes indicate genes present in the pathway but not detected in the data. Arrows show the connections between the genes and their interactions in the pathway. (TIF) [file pone.0318185.s003.tif]

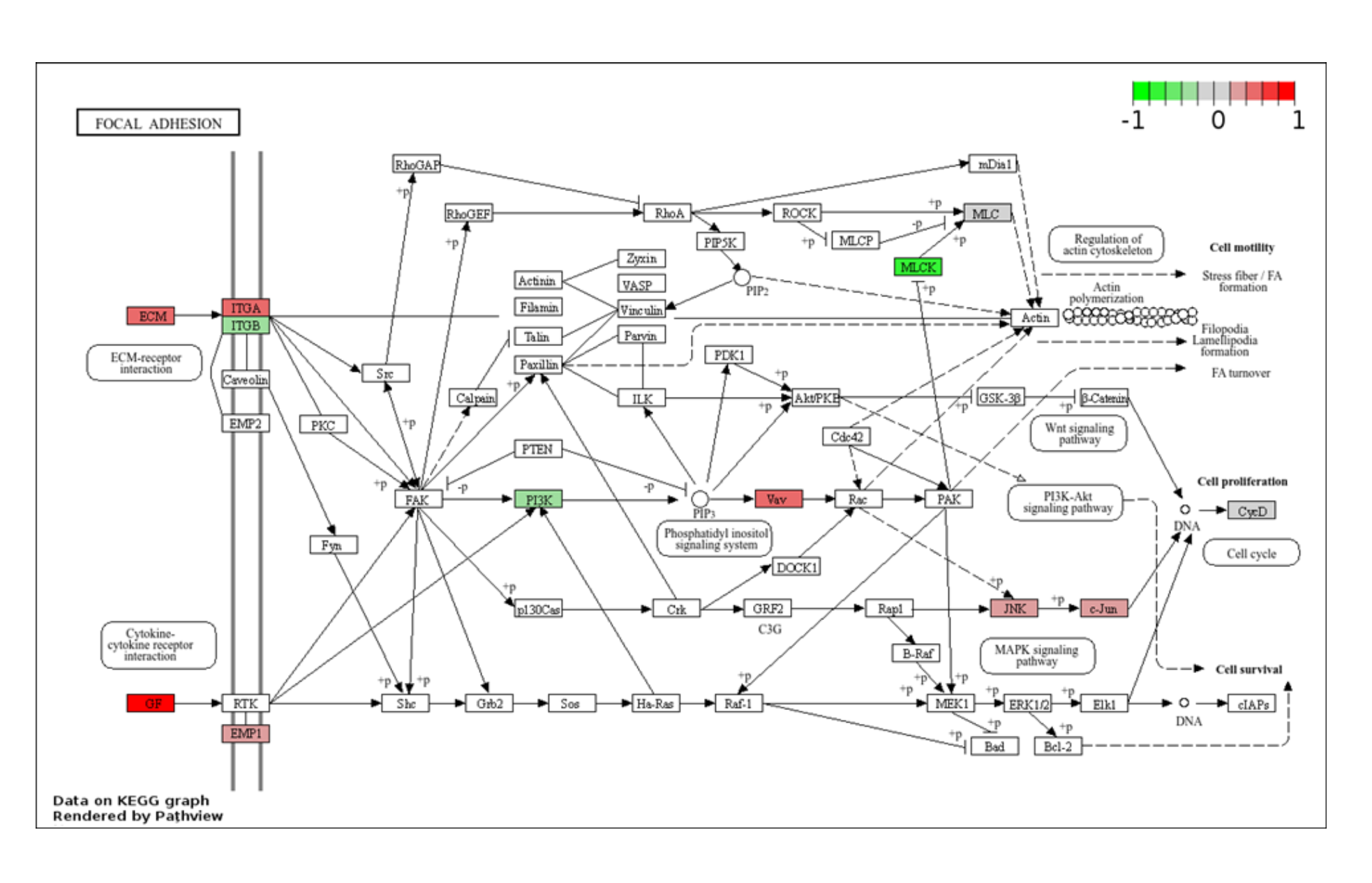

Supplement: S4 Fig — Focal adhesion pathway in response to 25 and 50 μM TQ treatment for 48 hours in A172 glioblastoma cells. Red boxes represent upregulated genes, while green boxes show downregulated genes. The intensity of the color reflects the level of change. Gray boxes indicate genes present in the pathway but not detected in the data. Arrows show the connections between the genes and their interactions in the pathway. (TIF) [file pone.0318185.s004.tif]

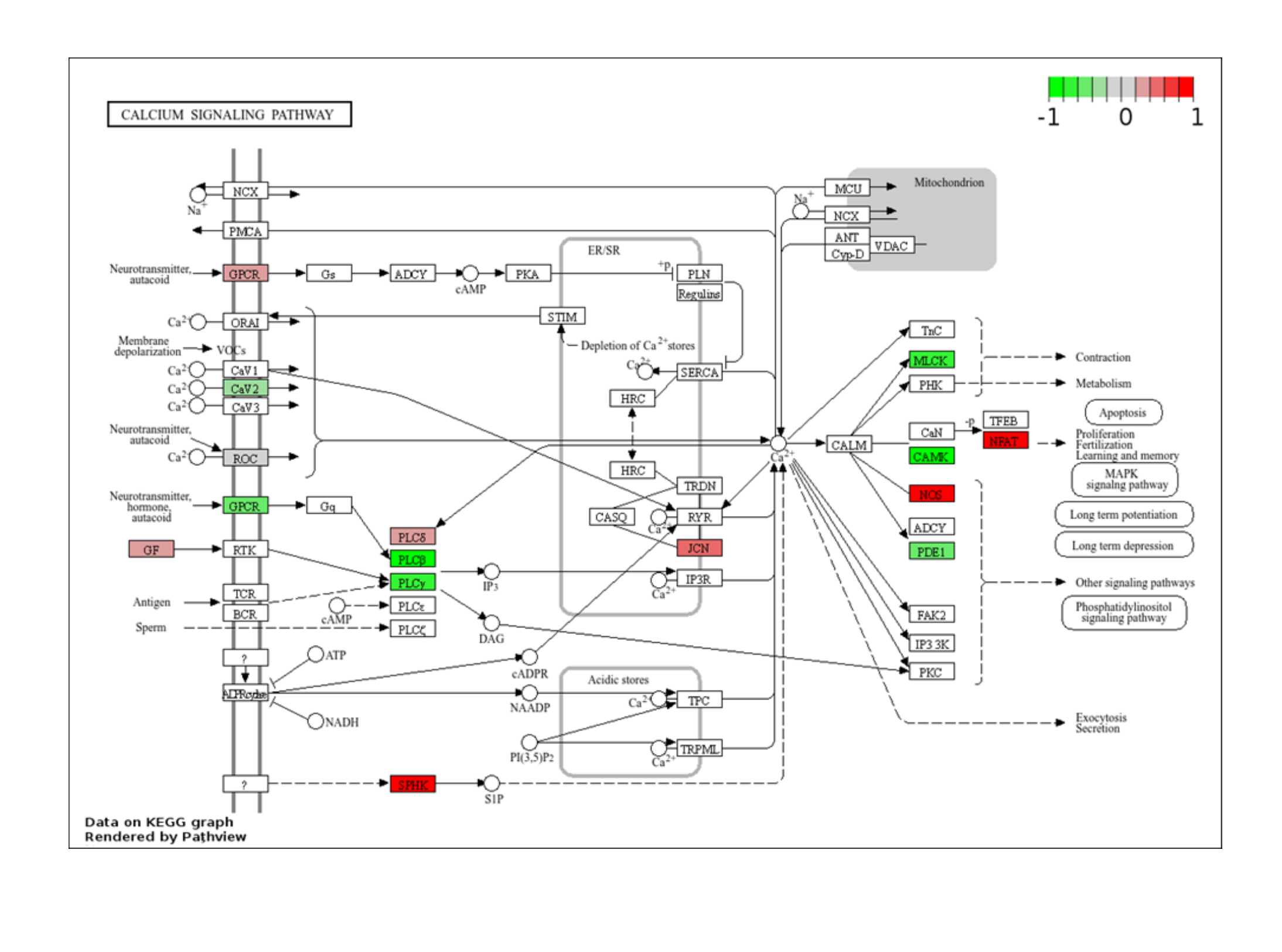

Supplement: S5 Fig — Calcium signaling pathway in response to 25 and 50 μM TQ treatment for 48 hours in A172 glioblastoma cells. Red boxes represent upregulated genes, while green boxes show downregulated genes. The intensity of the color reflects the level of change. Gray boxes indicate genes present in the pathway but not detected in the data. Arrows show the connections between the genes and their interactions in the pathway. (TIF) [file pone.0318185.s005.tif]

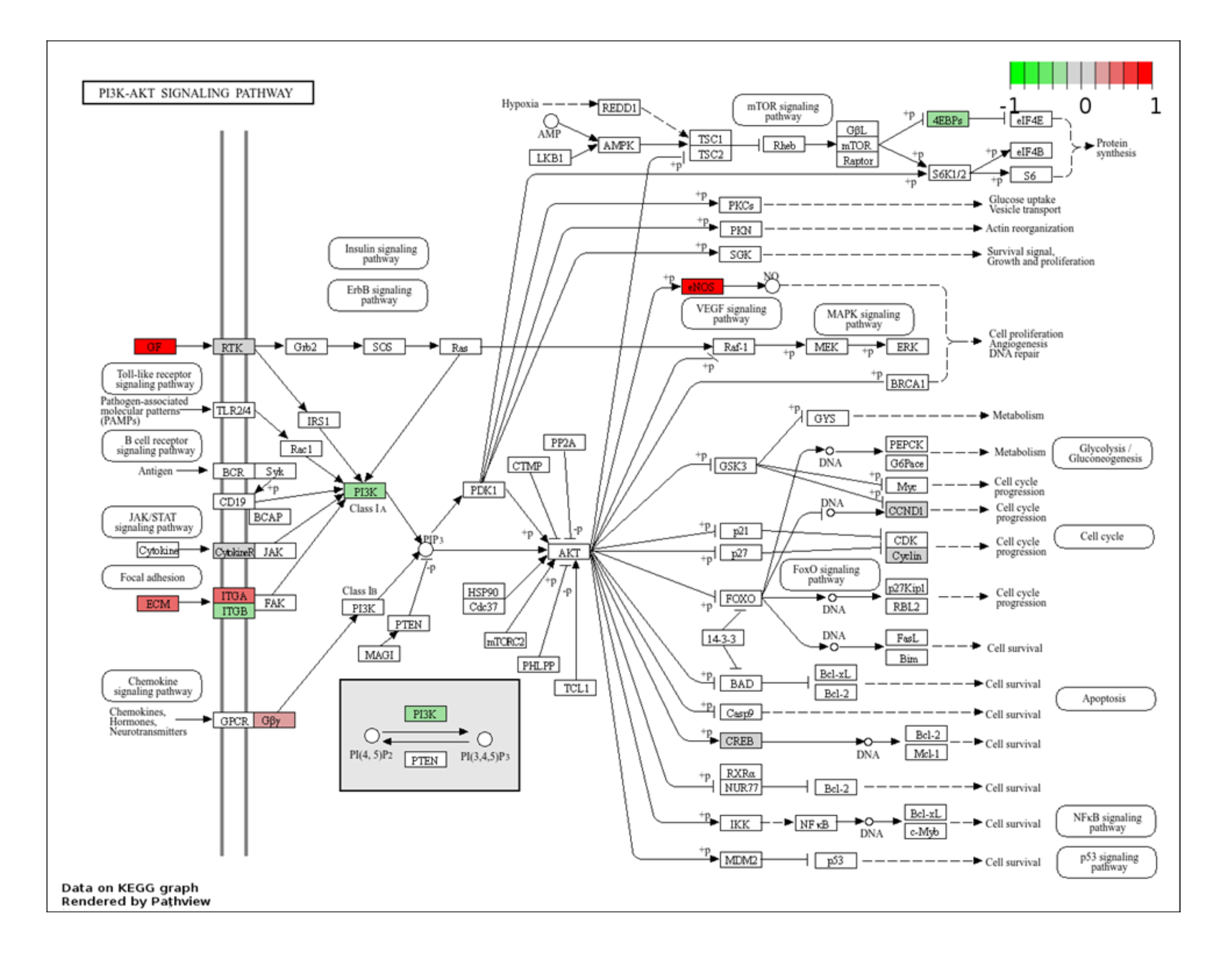

Supplement: S6 Fig — PI3K-AKT signaling pathway in response to 50 μM TQ treatment for 48 hours in A172 glioblastoma cells. Red boxes represent upregulated genes, while green boxes show downregulated genes. The intensity of the color reflects the level of change. Gray boxes indicate genes present in the pathway but not detected in the data. Arrows show the connections between the genes and their interactions in the pathway. (TIF) [file pone.0318185.s006.tif]

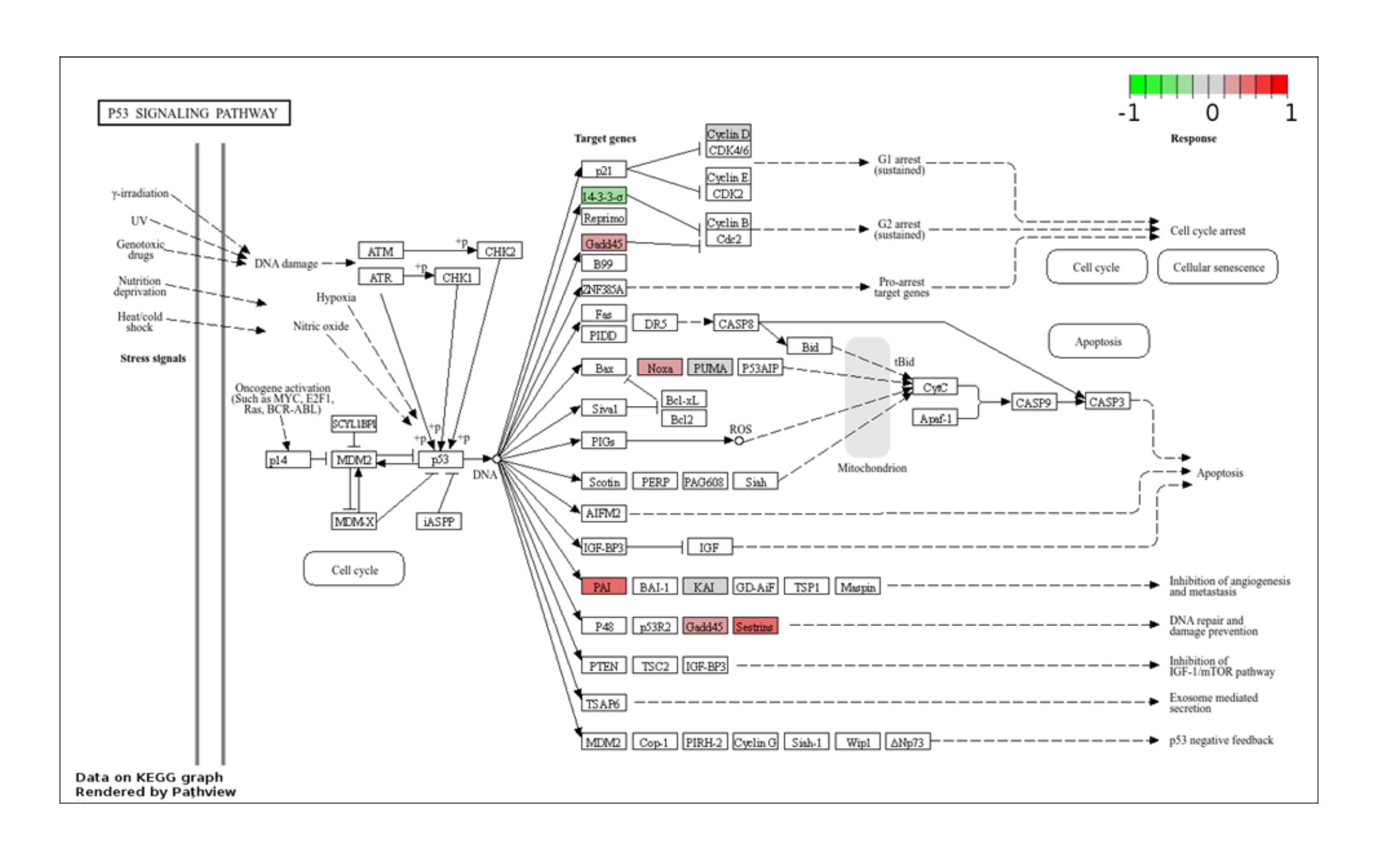

Supplement: S7 Fig — P53 signaling pathway in response to 25 and 50 μM TQ treatment for 48 hours in A172 glioblastoma cells. Red boxes represent upregulated genes, while green boxes show downregulated genes. The intensity of the color reflects the level of change. Gray boxes indicate genes present in the pathway but not detected in the data. Arrows show the connections between the genes and their interactions in the pathway. (TIF) [file pone.0318185.s007.tif]

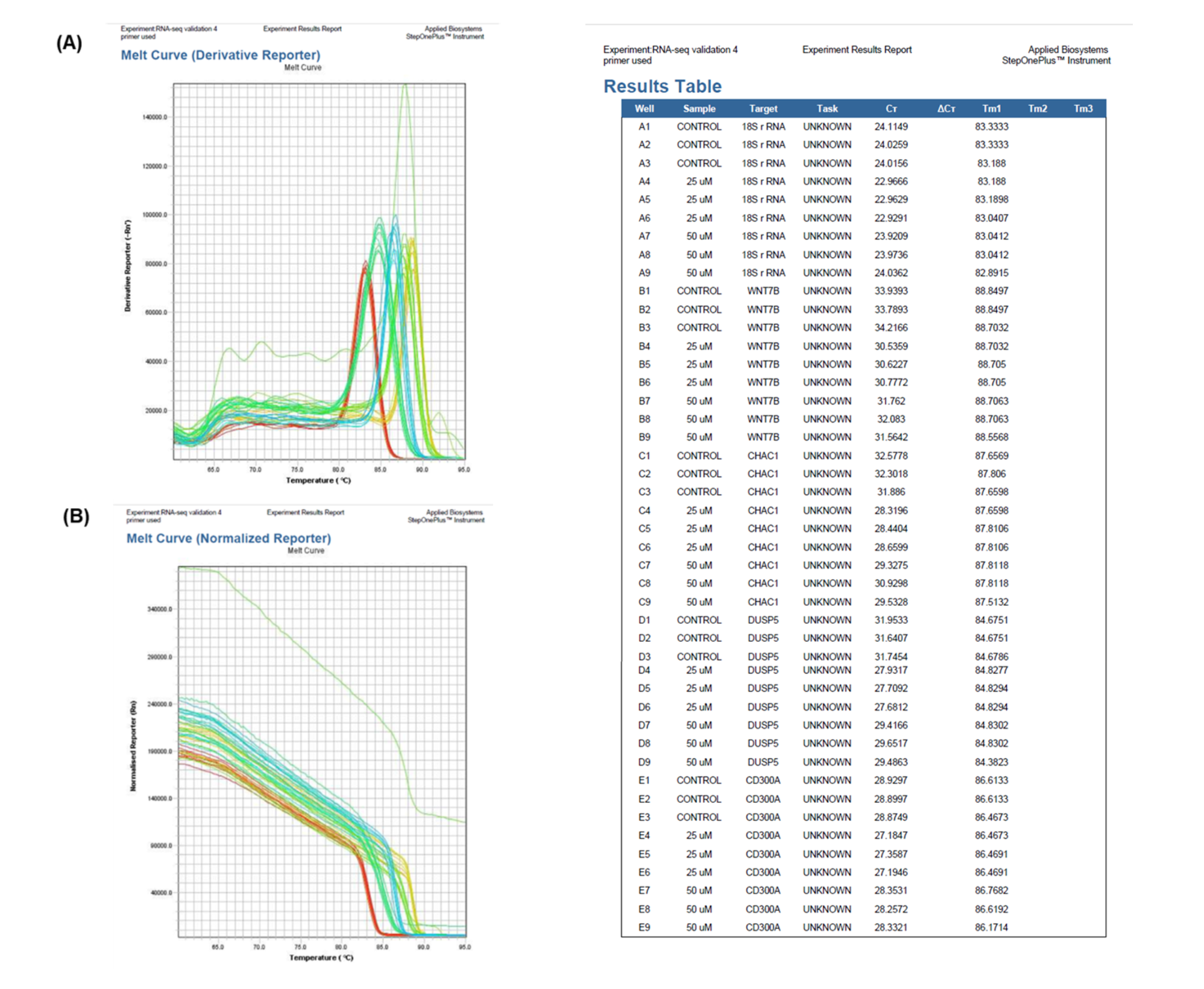

Supplement: S8 Fig — (A) Melt curve (derivative reporter) displaying amplification specificity for the housekeeping gene 18s rRNA (red) and the genes of interest: DUSP5 (green), CD300A (cyan), CHAC1 (light green), and WNT7B (yellow). The sharp peaks indicate single, specific amplicons for each gene, confirming specificity. (B) Melt curve (normalized reporter) showing fluorescence intensities normalized across the samples for consistent data interpretation. The normalized curves reaffirm the specificity of the amplified products. (C) Results table summarizing the qRT-PCR data, including Ct values, fold changes, and melting points (Tm) for each gene under different conditions (control, 25 μM, and 50 μM TQ treatments). Housekeeping gene 18s rRNA served as internal control. The melting temperatures for each gene align with their respective peaks in the melt curve analyses, confirming assay efficiency and specificity. (TIF) [file pone.0318185.s008.tif]
